# A Case of Morbus Coxarius, with Interesting and Unusual Complications

**Published:** 1857-09

**Authors:** O. C. Gibbs

**Affiliations:** Frewsburg, Chautaque Co., N. Y.


ART. III.—*A Case of Morbus Coxarius, with interesting and unusual Complications.* By O. C. Gibbs, M. D., Frewsburg, Chaut. Co., N. Y.

August 1st, 1853, I was called to see A. Young, aged 18 years, and found him laboring under considerable fever, with pain in the left leg, which the friends considered rheumatic. The pain extended throughout the whole limb, but was more severe in the knee, hip and ankle articulations. The patient had been engaged at work in the hay-field quite laboriously, being in unusual health, until about three days previous. He had a sore at the outer ankle, which was considerably inflamed and quite painful, which the patient thought originated in a gall made by his boot. The first sensation of pain was felt in the ankle, and the patient thought he had taken told in the abrasion, caused by his boot, developing rheumatic pains in the whole limb, to which form of disease (rheumatism) he averred he was partially subject. His position was, by choice, upon his back, with the limb drawn up, and, after a careful examination, I found that movements of the hip-joint, and particularly a blow upon the knee, driving the head of the femur upward against the acetabulum, was productive of much increase of pain.

In view of all the circumstances, which it is not necessary to further detail here, notwithstanding the recent manifestations of the disease and the affection at the ankle, of which the patient was most disposed to complain, the case was diagnosed as one of synovial, or cartilaginous inflammation of the hip-joint.

As the patient was decidedly scrofulous, and as the febrile symptoms were by no means of a decided sthenic character, active antiphlogistic measures were not resorted to. Blisters were applied about the hip, and repeated as often as circumstances would permit; mercurial ointment, for a time, was rubbed into the groin; saline evacuants, and the iodide of potassium, were the only internal medicines administered in the early period of the disease.

After three or four weeks, chronic abscesses began to form in different parts of the body, appearing without any evidences of inflammation, and healing readily on the evacuation of the pus which they contained. The head, face, neck, body, and extremities, were subject to these abscesses; the limbs perhaps more so than any other part. The pus, in some instances, would burrow under the muscles, and at one time, the probe could be passed from an opening at the middle of the fore arm, down to the wrist and up to the elbow; also, from one in the leg, down to the ankle and up to the knee. Seldom more than two or three appeared at the same time, but continued, one after another, until between eighty and ninety in all were opened, discharging pus, in amount, varying from a teaspoonful to seven or eight ounces. Fortunately they were all superficial; the internal organs remaining exempt from these accumulations of pus.

During this period, various alteratives and tonics were given: as for instance, cod liver oil, the various preparations of iron, including the iodide, the bi-bromide, &c., iodide of potassium with syrup of sarsaparilla, quinine, &c., not all at once, but in succession and variously combined.

During this period, the hip presented the symptoms usual in morbus coxarius, evidences of suppuration manifested themselves, and about the middle of October, an opening was made into the cavity about the joint, a pint of pus evacuated, and the cavity injected with a solution, containing one part tincture of iodine and two parts water. The pus was withdrawn every or every other day, and the cavity injected, for several weeks, and subsequently less frequently with various injections to be mentioned hereafter.

Soon after the opening was made into the collection of pus about the joint, a spontaneous opening appeared in the lumbar region, on the left of the spinal column, and still later one on the right, both remaining open, constantly discharging pus, except when prevented by a compress and adhesive straps.

From the opening in the left lumbar region, a probe could be passed down upon the inner surface of the ilium, and an injection thrown into either of the lumbar openings, could be forced through this sinus in front of the ilium to the cavity connected with the joint, and out at either of its two openings.

Under the influence of these, and the many superficial abscesses previously referred to, notwithstanding the persevering use of tonics and nourishing diet, the patient rapidly emaciated, and his vital forces dwindled, under the influence of the exhausting discharges, to the lowest ebb compatible with the maintenance of life. Apthæ appeared in the mouth and fauces, accompanied with cough and difficult respiration, and with tenacious, frothy mucous expectoration, as in capillary bronchitis. At this stage of the difficulty syrup of senega, with carbonate of ammonia, was given; also, the chlorate of potash in connection with stimulants and tonics. In passing, permit me to say that I have seen the chlorate of potash apparently operate most happily in some cases, with symptoms as above.

Though the patient's death was hourly expected, for two or three days, yet the pectoral symptoms gradually passed away and the patient began to improve. Though he did not gain flesh, yet he gradually returned to a tolerable degree of health; the sinus remained open about the hip and in the lumbar region, discharging less freely than at first, yet quite considerably. He remained thus comfortable through the winter and spring. During the summer and fall he was able to be taken from his bed and placed in a carriage, and to ride out every day, or less frequently, as suited his inclinations.

From extreme emaciation, or from spinal disease, he had but little control over his lower extremities, yet he could sit for a time, when placed in an erect position, and he would often amuse himself by shooting at a mark, or at birds upon the fruit trees, from his window.

The tonic and alterative plan of treatment was steadily pursued, with such variations in particular remedies, as circumstances seemed to justify. The sinuses were now injected only every second or third day, and tincture of iodine, tincture of myrrh, muriated tincture of iron, solutions of nitrate of silver, and sulphate of zinc, were all made use of, and with but little seeming difference in comparative results.

After the first three or four months of illness, he became considerably afflicted with gravel, occasionally passing stones varying in size from that of a kernel of wheat to a large sized pea. Once I was obliged to remove from the urethra, with slender forceps, one lodged there, which he had not the power to pass or dislodge.

During the summer and fall of 1854, he had occasional attacks of bilious vomiting; these would return, with tolerable regularity, once in ten days or two weeks. Various remedies were tried to prevent their recurrence, without effect, until I commenced giving pills of extract of wahoo, two or three times a week; after which, these evidences of bilious derangement ceased to trouble the patient.

During the spring of 1855, the sinuses extended and formed openings at the lower portion of the ischium, and also near the margin of the anus. After these openings were formed, in consequence of their position, they formed the principal outlets for the pus, and being considerably inflamed and painful, they prevented any further attempts at sitting up or riding out.

From this date, he remained much the same through the summer, fall, and winter of 1855. The left leg was much shortened, the hip-joint immovable, and the knee bent at right angles, and fixed there in consequence of the rigidity of the muscles. In consequence of his position in bed, which was toward the right side, the chest became misshaped, and the heart dislodged from its natural position.

February 20th, 1856, he was taken with a severe chill, and for a few days previously he had been troubled with cough and difficult breathing. The chills recurred daily, and the gravity of the pectoral symptoms increased until the 25th, when he died.

I very much regret to say that no post-mortem was allowed.

From the dates, it will be seen that the patient lived more than two and a-half years after the attack, most of that time under the depressing influence of constant and exhausting purulent discharges.

There is one fact connected with this case which I have neglected to mention, which may and may not have had some connection with the origin of this difficulty. The patient, from childhood, had been afflicted with occasional paroxysms of asthma. Some person had told him that urine would relieve the paroxysms, and, if persisted in, ultimately eventuate in a cure; consequently, during the spring and summer preceding his attack, he had been in the habit of taking nightly, on going to bed, a draught of his own urine!

It is surprising to observe how indifferent persons sometimes are to judicious prescriptions, yet with what commendable perseverance they will take a filthy dose, when advised by some illegitimate and unreliable prescriber.

It is highly probable that the uric acid had much to do with the development of that tendency to the formation of chronic abscesses, with which morbus coxarius and the psoas disease had more or less connection, ultimating eventually in death.

